# La soledad como predictor de mortalidad en pacientes con cáncer, un estudio de cohorte

**DOI:** 10.7705/biomedica.7150

**Published:** 2024-05-31

**Authors:** Adriana Valdelamar, Fernando de la Hoz, Ricardo Sánchez

**Affiliations:** 1 Grupo del Área de Investigación Clínica y Epidemiológica del Cáncer, Instituto Nacional de Cancerología ESE, Bogotá, D.C., Colombia Instituto Nacional de Cancerología ESE Instituto Nacional de Cancerología ESE Bogotá, D.C. Colombia; 2 Grupo de Epidemiología y Evaluación en Salud Pública, Facultad de Medicina, Universidad Nacional de Colombia, Bogotá, D.C., Colombia Universidad Nacional de Colombia Universidad Nacional de Colombia Bogotá, D.C. Colombia; 3 Departamento de Salud Pública, Facultad de Medicina, Universidad Nacional de Colombia, Bogotá, D.C., Colombia Universidad Nacional de Colombia Universidad Nacional de Colombia Bogotá, D.C. Colombia; 4 Instituto de Investigaciones Clínicas, Facultad de Medicina, Universidad Nacional de Colombia, Bogotá, D.C., Colombia Universidad Nacional de Colombia Universidad Nacional de Colombia Bogotá, D.C. Colombia

**Keywords:** soledad, neoplasias, mortalidad, aislamiento social, estudios de cohortes, salud pública., Loneliness, neoplasm, mortality, social isolation, cohort studies, public health.

## Abstract

**Introducción.:**

Algunos estudios han señalado que la soledad podría estar relacionada con un aumento en el riesgo de mortalidad en pacientes con cáncer ya que puede debilitar la respuesta al tratamiento y del sistema inmunológico y promover comportamientos perjudiciales, lo que puede empeorar el pronóstico y aumentar la probabilidad de muerte en estos pacientes. El abordar la soledad en la salud pública es esencial para brindar apoyo social y mejorar los resultados en los pacientes con cáncer.

**Objetivo.:**

Obtener un estimador de la asociación soledad no deseada - mortalidad en pacientes con cáncer.

**Materiales y métodos.:**

Se le hizo el seguimiento durante dos años a una cohorte prospectiva de 400 pacientes (exposición=niveles de soledad; desenlace=tiempo hasta la muerte). Se incluyeron variables de control sociodemográficas y clínicas. Se utilizó un modelo de supervivencia paramétrico (log normal).

**Resultados.:**

En la cohorte se encontró una mediana de supervivencia de 20,2 meses y una tasa de mortalidad de 3,2 muertes por 100 pacientes-mes (IC_95%_: 2,8 a 3,7). En el modelo de supervivencia se encontraron las siguientes razones de tiempo (RT): nivel moderado-nivel bajo: RT=0,55; IC_95%_: 0,39 a 0,77; nivel moderadamente alto-nivel bajo: RT=0,62; IC_95%_: 0.41 a 0.93; nivel alto-nivel bajo: RT=1,17; IC_95%_: 0,31 a 4,42.

**Conclusión.:**

En comparación con los pacientes con niveles bajos de soledad, los pacientes con niveles moderados o moderadamente altos llegan más rápidamente a la muerte (RT estadísticamente significativas, habiendo ajustado por el efecto de las demás variables del modelo). Esto sugiere la utilidad de las intervenciones para mitigar la soledad y promover el apoyo social en los pacientes con cáncer.

## Abstract (en)

**Introduction.:**

Some studies have indicated that loneliness may be associated with an increased risk of mortality in cancer patients, as it can weaken treatment response and the immune system, and promote harmful behaviors, worsening the prognosis and increasing the likelihood of death. Addressing loneliness in public health is essential to provide social support and improve outcomes in cancer patients.

**Objective.:**

To obtain an estimator of the unwanted loneliness-mortality association. Materials and methods. We followed a prospective cohort of 400 patients for two years (exposure=loneliness levels; outcome=mortality; sociodemographic and clinical control variables were included). A parametric survival model (log normal) was used.

**Results.:**

The cohort had a median survival of 20.2 months and a mortality rate of 3.2 deaths/100 patient-months (95% CI: 2.8 to 3.7). The survival model found the following time ratios (TR): moderate level/low level: TR=0.55; 95% CI: 0.39 to 0.77; moderately high level/ low level: TR=0.62; 95% CI: 0.41 to 0.93; high level/low level: TR=1.17; 95% CI: 0.31 to 4.42.

**Conclusion.:**

Compared to patients with low levels of loneliness, patients with moderate or moderately high levels reach death more quickly (statistically significant TRs, adjusted for the effect of other variables in the model); this highlights the need for interventions to mitigate loneliness and promote social support in patients having cancer.

El cáncer ocupa el segundo lugar como causa de muerte a nivel mundial, y solo en el año 2022 se registraron 9,74 millones de fallecimientos debido a esta enfermedad. Se estima que esta cifra aumentará a 16,9 millones para el año 2045 ^1^. En cuanto a la mortalidad, en Colombia se registraron 56.700 muertes por cáncer, aproximadamente, siendo más alta en hombres que en mujeres ^2^^,^^3^. Estas cifras resaltan la magnitud del problema del cáncer como una preocupación significativa para la salud pública, con un impacto notable en la morbimortalidad de la población ^4^^,^^5^.

A medida que aumenta la incidencia del cáncer, también se observa un incremento en el número de personas que sobreviven a esta enfermedad. Sin embargo, tanto los pacientes como los supervivientes a menudo enfrentan desafíos en su calidad de vida y bienestar general, ya que esta enfermedad se ha relacionado con factores psicosociales, entre los que se han destacado la depresión y el pobre apoyo social ^6^^-^^8^, los cuales también se han relacionado, no solo con su aparición, sino con su progresión y gravedad ^9^.

A pesar de los avances tecnológicos y los hallazgos relacionados con los factores que influyen en la mortalidad del cáncer, todavía existe un vacío en el conocimiento sobre los factores o determinantes que aceleran la mortalidad en pacientes diagnosticados con esta enfermedad, especialmente aquellos relacionados con aspectos psicosociales, emocionales y sociales ^10^^-^^12^.

Estos factores psicosociales y emocionales pueden desempeñar un papel significativo en la experiencia del paciente y su respuesta al tratamiento, y su comprensión y abordaje adecuados son cruciales para mejorar los resultados y la calidad de vida ^13^^,^^14^. Un ejemplo de esto es la soledad no deseada, un constructo que ha sido definido por varios autores como la percepción subjetiva de aislamiento y falta de conexión emocional con los demás, a pesar de desear y buscar activamente relaciones sociales significativas ^15^^-^^18^.

Es importante distinguir entre los conceptos de soledad y aislamiento, dado que este último se refiere a la ausencia objetiva o escasez de contactos e interacciones dentro de una red social ^19^. Esta distinción adquirió una relevancia particular durante la pandemia de COVID-19, ya que muchas personas pudieron tener menos contactos, pero no todas experimentaron sensaciones de soledad. Por el contrario, algunas personas podrían encontrarse rodeadas de un número considerable de individuos, pero aun así sentirse solas y desconectadas. Esto podría deberse a que la soledad está influenciada por otros factores que van más allá del simple aislamiento social, como son las características del individuo y su entorno estables en el tiempo, tales como rasgos de personalidad, necesidades de contacto y expectativas respecto a relaciones saludables tanto físicas como mentales, así como normas culturales, según se menciona en una revisión sistemática ^20^.

Para medir este constructo, se han propuesto diversos instrumentos, entre los que se destacan la Escala de Soledad de UCLA, ampliamente utilizada ^21^, la Escala de Soledad Emocional (SELSA-S) ^22^ y la Escala de Soledad Social ^23^ entre otras. Estas escalas han demostrado tener propiedades psicométricas adecuadas y han sido utilizadas en diversos estudios observacionales ^24^.

Por otro lado, varios estudios han establecido una relación entre la soledad no deseada y un mayor riesgo para la salud física y mental, particularmente en pacientes con enfermedades crónicas como es el cáncer ^25^. La soledad no deseada puede afectar el funcionamiento inmunológico, generar mayor fatiga, dolor, trastornos del sueño y alteraciones emocionales, lo que empeora la condición de salud y afecta la calidad de vida ^26^. Además, se ha observado que la soledad no deseada puede estar asociada a un mayor grado de mortalidad en diferentes tipos de enfermedades como la enfermedad cardiovascular, la respiratoria, el cáncer o por cualquier otro tipo de causa ^27^^-^^29^ e incluso, ha sido comparada con factores de riesgo para la salud como el tabaquismo o la obesidad ^30^ y se ha observado que la soledad está relacionada con un aumento en la mortalidad por diversas causas tanto en hombres como en mujeres.

Sin embargo, este efecto parece ser ligeramente más pronunciado en hombres. Una posible explicación de esta diferencia podría radicar en que los hombres, debido a influencias culturales, pueden ser más reticentes a admitir sentirse solos y es posible que informen sentirse solos cuando la intensidad de este sentimiento es alta, lo que resulta en un impacto más significativo ^31^^,^^32^.

Aun cuando algunas investigaciones han planteado los efectos negativos de la soledad no deseada en los pacientes con cáncer y se ha asociado con muerte prematura, son pocos los estudios longitudinales que han establecido la relación entre soledad y mortalidad en este grupo de pacientes; uno de ellos es el de Liang Fu You (2014), con un seguimiento de cinco años, en el que no solo se evaluó epidemiológicamente dicha asociación, sino que también se validaron 34 genes asociados a la soledad que tenían una buena predicción de supervivencia para los pacientes con cáncer de hueso (HR =5,10; p < 0,001), cáncer de pulmón (HR = 2,86, p < 0,0001), cáncer de ovario (HR = 1,97, p < 0,0001), leucemia (HR = 2,06, p < 0,0001) y linfoma (HR = 3,50, p< 0,0001) ^33^.

Otro estudio, realizado en población sueca (N = 1363), determinó que la soledad se asoció con un aumento significativo del riesgo de mortalidad del 27 % en comparación con las personas que no se sentían solas (HR = 1,27; IC_95%_: 1,01-1,60), lo cual sugiere una asociación entre la soledad y un mayor riesgo de mortalidad ^34^. Resultados similares se obtuvieron en otro estudio prospectivo en el que la soledad predijo la mortalidad; sin embargo, en el grupo de pacientes con cáncer, la asociación entre soledad y mortalidad se perdió si se ajustaba por variables relacionadas con depresión y estilo de vida ^35^.

Sin embargo, la mayoría de la literatura científica revisada sobre la relación entre la soledad y la mortalidad se ha enfocado en países de altos ingresos como Estados Unidos, Reino Unido, Japón, España, Suecia y China, donde se ha observado una notable transición sociodemográfica hacia el envejecimiento, una alta esperanza de vida y una mayor incidencia de cáncer ^30^^,^^36^.

Es importante destacar que los resultados de los estudios sobre la soledad, incluso antes del surgimiento de la pandemia de COVID-19, estaban emergiendo como preocupaciones cruciales tanto en el ámbito político como en el de la salud pública. Esto se debe en gran parte a su profundo impacto en la longevidad, así como en la salud física, mental y el bienestar en general. La llegada de la pandemia y las medidas subsiguientes para contenerla han intensificado el estudio de los problemas relacionados con la soledad. Durante la pandemia y después de ella, se han llevado a cabo investigaciones que han abordado específicamente la soledad y su influencia en los pacientes con cáncer. Estos estudios han arrojado resultados variados en cuanto a los efectos de la pandemia en los niveles de soledad y su correlación con el deterioro de la salud mental ^37^^-^^39^.

Teniendo en cuenta este contexto, el presente estudio tuvo como objetivo explorar la relación entre la soledad y la mortalidad en pacientes con cáncer a través de una cohorte prospectiva de pacientes tratados en un centro oncológico de referencia nacional en Bogotá (Colombia), durante el período comprendido entre el 2020 y el 2023, en el contexto de la pandemia de COVID-19. Dada la situación de pandemia, un grupo de mediciones se llevó a cabo mediante encuestas telefónicas, siguiendo un enfoque similar al utilizado en otros estudios documentados en la literatura ^20^^,^^37^^,^^39^.

## Materiales y métodos

### Tipo de estudio

El presente es un estudio de cohorte prospectiva desarrollado durante los años 2020 a 2023 en el Instituto Nacional de Cancerología, en el que se tomó como desenlace el tiempo hasta la muerte por cualquier causa y como exposición los niveles de soledad medidos con la escala de soledad de UCLA, versión 3.

La literatura aún sigue siendo escasa y no muestra información consistente sobre el posible efecto de la soledad, o sobre el mayor o menor impacto en relación con diferentes localizaciones de cáncer o con el estadio de la enfermedad ^40^.

### Población de estudio

Se ubicó en los registros clínicos de pacientes mayores de 18 años, que estuvieran en proceso de diagnóstico o tratamiento activo en el Instituto Nacional de Cancerología y que tuvieran diagnóstico confirmado histopatológicamente de alguna de las cuatro localizaciones diferentes de cáncer con alta prevalencia en Colombia: mama, próstata, cuello uterino y gastrointestinal. Es importante destacar que la población de pacientes perteneciente al Instituto Nacional de Cancerología está constituida principalmente por personas de estratos socioeconómicos medios y bajos, provenientes de diversas regiones del país y mayoritariamente afiliados al régimen subsidiado de salud. Además, existe una notable presencia de casos en estadios avanzados de la enfermedad ^41^.

En este contexto, los pacientes con las anteriores características fueron contactados en los servicios de consulta externa de la institución y se les realizó una entrevista inicial en la que se verificaron los criterios de elegibilidad y se confirmó el consentimiento para participar en el estudio. Adicionalmente, dependiendo de la disponibilidad del paciente, se efectuó una medición inicial de los niveles de soledad usando la escala de soledad de UCLA, versión 3 (UCLA 3); la encuesta fue aplicada por dos profesionales de salud con experiencia de 6 años en la utilización de instrumentos para la medición de los desenlaces reportados por los pacientes. Si los pacientes no tenían disponibilidad de tramitar las escalas en ese mismo momento, se efectuaba una programación para realizar las mediciones mediante aplicación de las escalas por vía telefónica en un plazo no mayor de cinco días luego del contacto inicial.

Esta medición de soledad se continuó realizando cada seis meses, de manera presencial o telefónica. Para cada paciente se planteó un seguimiento mínimo de dos años. Durante el período de aislamiento relacionado con la pandemia de COVID-19, la aplicación de las escalas se efectuó telefónicamente, de forma mensual. Luego de la primera aplicación de la escala UCLA 3, se comprobaba el estatus vital del paciente mediante verificación de su asistencia ya fuera por teleconsulta o presencialmente en los controles ambulatorios verificando las historias clínicas del sistema SAP, o mediante llamada telefónica.

En caso de fallecimiento del paciente, se registraba la fecha exacta de este evento. Las variables clínicas y de tratamiento fueron tomadas de los registros clínicos institucionales. La información relacionada con las variables del estudio se registró y administró en una base de datos diseñada en el programa REDCap.

El presente estudio forma parte del proyecto titulado “Validación de la Escala de Soledad de UCLA y la Escala de Soledad en Cáncer en pacientes con cáncer en la población colombiana”. El proyecto fue aprobado en el acta del 18 de junio de 2019 por el comité de ética en investigación del Instituto Nacional de Cancerología.

### Variables del estudio

#### Variable de desenlace

Se tomó como variable de desenlace la mortalidad por cualquier causa. Como se mencionó previamente, esta variable se evaluó mediante el seguimiento mensual a los pacientes para determinar su estatus vital. Este seguimiento se practicó durante dos años luego de la inclusión en el estudio. La pérdida del seguimiento podía darse por imposibilidad de poder volver a contactar al paciente en algún momento; en caso de fallecimiento, se registró la fecha de la muerte, y en caso de pérdida de seguimiento, se registró la fecha del último contacto con el paciente en el que se pudo verificar que no había fallecido. Los eventos de pérdida de seguimiento y los de final de seguimiento por terminación del estudio se manejaron en los análisis como censuras a la derecha, y los fallecimientos como desenlaces.

### Variables independientes

#### Soledad no deseada

Esta variable se tomó como principal variable independiente de nuestro estudio. La medición del constructo se realizó con la escala de soledad de UCLA, versión 3 ^42^, que cuenta con una versión traducida y adaptada transcultural mente para ser utilizada en Colombia ^43^. Se seleccionó este instrumento debido a su amplia utilización en investigaciones relacionadas con la salud, así como por sus satisfactorias propiedades de medición informadas en diferentes estudios de validación. Estos estudios reportaron un coeficiente alfa de Cronbach que osciló entre 0,89 y 0,93 y una confiabilidad test-retest durante un período de un año de 0,73 ^24^^,^^44^. Además, se ha establecido su validez convergente a través de correlaciones significativas con otras medidas de soledad ^24^.

La puntuación total en la escala varía de 20 a 80, donde las puntuaciones más altas indican mayor soledad. La categorización más comúnmente utilizada se divide en cuatro niveles: puntajes entre 20 y 34 denotan un grado bajo de soledad; entre 35 a 49, un grado moderado de soledad; entre 50 y 64, un grado moderadamente alto de soledad, y entre 65 y 80, un alto grado de soledad ^24^. Además, la UCLA, versión 3, se ha utilizado como una medida unidimensional de soledad, conceptualizando y evaluando la soledad como una experiencia global. Sin embargo, se ha propuesto una perspectiva multidimensional de la soledad, que considera varios aspectos de la experiencia de soledad, como la soledad relacionada con otros íntimos, otros sociales y el entorno afiliativo ^45^.

Haciendo la búsqueda de la literatura encontramos que la mayoría de los estudios de validación de esta escala se han llevado a cabo en población general o con otros tipos de enfermedad y no se han encontrado estudios de validación en pacientes con cáncer. Para el presente estudio se utilizó la versión de la escala UCLA 3 adaptada transculturalmente y validada en población con cáncer en Colombia, que mostró una estructura unidimensional, adecuados valores de validez divergente, concurrente y predictiva, así como satisfactorios coeficientes de confiabilidad (omega de McDonald’s = 0,94; coeficiente de correlación-concordancia de Lin > 0,8) ^46^. En este estudio, para el modelo de regresión, se utilizó la categorización de la escala en cuatro niveles, como se planteó en un estudio previo ^24^.

### Variables sociodemográficas

Dentro de este grupo de variables se incluyeron el sexo (masculino o femenino), la edad del paciente medida en años, el nivel de escolaridad y el estrato socioeconómico en el momento de la inclusión al estudio.

### Variables clínicas

En este grupo se incluyeron variables con potencial influencia sobre la mortalidad; con este criterio se seleccionaron las siguientes:

*Localización del cáncer:* se tomaron localizaciones de cáncer con prevalencias altas, correspondientes a tumores sólidos, de acuerdo con las siguientes localizaciones: mama, próstata, cuello uterino y gastrointestinal (estómago y colorrectal).

*Estadio clínico:* se tomó la estadificación consignada en las historias clínicas electrónicas, confirmada después de reunión de junta multidisciplinaria en los distintos servicios de atención clínica (seno y tejidos blandos, ginecología, urología y gastroenterología).

*Tratamiento:* se consignó el tipo de tratamiento que estaba recibiendo el paciente durante el seguimiento. Se ubicaron cuatro grupos de diferentes tratamientos: cirugía, radioterapia, quimioterapia y cuidados paliativos.

*Comorbilidad:* para la medición de esta variable, se aplicó el índice de Charlson que permite cuantificar la comorbilidad que presenta un paciente. El índice asigna distintas ponderaciones a diferentes tipos de comorbilidad. La suma de dichas comorbilidades ponderadas genera un puntaje global de comorbilidad. Los puntajes más altos se han relacionado con mayor riesgo de mortalidad y de utilización de recursos de salud ^47^.

*Funcionalidad:* esta variable fue medida utilizando el índice de Karnofsky, utilizado para evaluar la capacidad de los pacientes con cáncer para realizar tareas rutinarias. Esta escala asigna puntajes que van de 0 a 100, donde un puntaje más alto indica una mayor capacidad del paciente para llevar a cabo actividades cotidianas. Además, puede emplearse para determinar el pronóstico del paciente y medir los cambios en su capacidad funcional a lo largo del tiempo ^48^.

### Análisis estadístico

El cálculo del tamaño de la muestra tuvo en cuenta dos componentes: uno relacionado con el cálculo de un estimador de la asociación entre niveles de soledad y riesgo de muerte, y el otro relacionado con la construcción de un modelo multivariable.

Para el primer componente se tuvieron en cuenta los siguientes insumos: para un HR de 1,4 ^49^, con valores de significación del 5 %, poder del 80 %, una proporción de fallecimientos en la cohorte de 0,35 ^50^ y un R^2^ para las variables independientes de 0,45 se requeriría un tamaño de muestra, al menos, de 375 pacientes ^51^.

Para el segundo componente, teniendo en cuenta la incorporación, aproximadamente, de 12 covariables (se incluyen en esta cuenta las variables indicadoras) se requiere un total de 120 desenlaces; si la frecuencia esperada de eventos es de 0,35 se requeriría una muestra, al menos, de 343 pacientes ^52^. Tomando el peor escenario (375 pacientes) y anticipando una posible pérdida de datos en los modelos multivariables por valores faltantes en alguna de las variables independientes, se sobreestimó este tamaño en un 5 % por lo cual se consideró un tamaño de muestra mínimo de 400 pacientes.

Para los análisis descriptivos se utilizaron medias y desviaciones estándar (DE) o medianas y rangos intercuartílicos (RIC), o frecuencias absolutas y porcentajes dependiendo del tipo de variable. La frecuencia de fallecimientos durante la cohorte se describió usando tasas de incidencia; este estimador fue reportado con intervalos de confianza del 95 %. Se calcularon y graficaron funciones de supervivencia usando el estimador de Kaplan-Meier; asimismo, se compararon las funciones de supervivencia de acuerdo con los cuatro estratos de niveles de soledad mencionados previamente ^25^ utilizando la prueba de log rank. Con los estimadores de Kaplan-Meier se calculó la mediana de supervivencia en la cohorte, la cual se reportó con el percentil 25 % (el percentil 75 no se alcanzó).

Para evaluar el efecto de la soledad no deseada habiendo ajustado por las variables sociodemográficas y clínicas previamente mencionadas se utilizó inicialmente un análisis de supervivencia usando un modelo de riesgos proporcionales de Cox. En dicho modelo se incorporó como covariable dependiente de tiempo el nivel de soledad no deseada, considerando que dicha variable se midió repetidamente cada seis meses; el uso de estas medidas repetidas ofrece una mejor información sobre la dinámica del constructo que una medición única.

**Figura 1.**
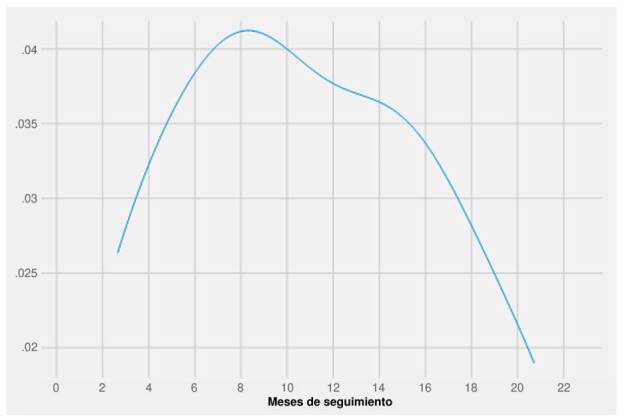
Estimación de la función de peligro suavizada con filtro gausiano

El supuesto de riesgos proporcionales se evaluó mediante una prueba de pendiente diferente de cero a partir de los residuales escalados de Schoenfeld ^53^. Para este modelo también se efectuaron diagnósticos de colinealidad y de valores influyentes. Ante la violación del supuesto de riesgos proporcionales se recurrió a un modelo paramétrico log normal; la selección de este modelo se hizo teniendo en cuenta las características de la función de peligro (figura 1) y los valores de los criterios de información de Akaike y bayesiano, que para el caso del modelo log normal fueron los más bajos entre cinco modelos comparados (Weibull, Gama, log normal, log logístico y exponencial). Las estimaciones del modelo log normal se presentan en la métrica de tiempo acelerado, también conocidos como modelos de tiempo acelerado (*Accelerated Failure Time*, AFT), usando razones de tiempo (RT) para facilitar su interpretación.

Se efectuó una estimación inicial del modelo crudo (tiempo hasta la muerte dependiente de nivel de soledad), utilizando la métrica de razones de peligro.

Para los modelos estadísticos se utilizaron hipótesis a dos colas y un nivel de significación del 5 %. Los análisis estadísticos fueron realizados con el programa Stata 16™.

## Resultados

Se aplicaron los criterios de elegibilidad en 473 pacientes, de los cuales se excluyeron 73 por los siguientes motivos:

- diagnóstico sospechoso y no confirmado al momento de la aplicación de la escala y al inicio del primer seguimiento (n = 15);
- imposibilidad de contacto por vía telefónica o personal debido a medidas de aislamiento obligatorio por el COVID-19 (n = 45), y
- decisión voluntaria de retirarse del estudio, falta de interés en participar en el seguimiento o suspensión de la aplicación de la escala (n =13).

Los 400 pacientes de la muestra se completaron seleccionando 100 por cada una de las localizaciones de cáncer previamente mencionadas mediante un muestreo no probabilístico, por conveniencia consecutivo hasta completar el tamaño de cada localización de cáncer.

La muestra estuvo conformada mayoritariamente por mujeres (64,5 %), con un predominio de estratos socioeconómicos bajos y niveles de escolaridad inferiores a ocho años. Menos del 2 % de los pacientes registró puntajes altos de soledad. Los 211 fallecimientos en la cohorte representaron una tasa de mortalidad de 3,2 muertes por 100 pacientes-mes (IC_95%_: 2,8 a 3,7). Las tasas de mortalidad según la localización del cáncer fueron las siguientes: gastrointestinal, 5,17 muertes por 100 pacientes-mes (IC_95%_: 4,09 a 6,53); próstata, 4,15 muertes por 100 pacientes-mes (IC_95%_: 3,25 a 5,29); cérvix, 2,56 muertes por 100 pacientes-mes (IC_95%_: 1 91 a 3 43), y mama, 1,61 muertes por 100 pacientes-mes (IC_95%_: 1,14 a 2,30). En comparación con los no fallecidos, los pacientes que fallecieron durante el seguimiento tenían mayor edad y comorbilidad. Hubo mayor porcentaje de muertes en el grupo de pacientes con cáncer gastrointestinal y de próstata, en los que tenían estadios avanzados y en los que recibieron tratamiento de cuidados paliativos. Los pacientes que fueron tratados con cirugía tuvieron menor frecuencia de fallecimientos. Las características sociodemográficas y clínicas evaluadas en el estudio se presentan en el cuadro 1.

**Cuadro 1.** Características de la muestra según desenlace de mortalidad

		Vivos	Fallecidos	Total
n		189	211	400
n Edad (media ± DE)*		57,8 ± 12,5	61,0 ± 13,2	59,5 ± 13
Puntaje total escala UCLA (media ± DE)		35,5 ± 11,6	35,3 ± 12	35,4 ± 11,8
Índice de Charlson [mediana (RIC)]*		6 (3 a 8)	8 (6 a 9)	7 (4 a 8)
Escala de Karnofsky [mediana (RIC)]		80 (70 a 90)	80 (70 a 90)	80 (70 a 90)
		n (%)	n%	n%
Puntaje UCLA categorizado				
	Bajo	100 (52,9)	118(55,9)	218(54,5)
	Moderado	61 (32,3)	58 (27,5)	119(29,8)
	Moderamente alto	26(13,8)	32(15,2)	58 (14,5)
	Alto	2 (1,1)	3 (1,4)	5 (1,3)
Estrato socioeconómico*				
	1	55 (29,6)	46 (21,9)	101 (25,5)
	2	76 (40,9)	112(53,3)	188 (47,5)
	3	44 (23,7)	48 (22,9)	92 (23,2)
	4	11 (5,9)	4 (1,9)	15 (3,8)
Sexo*				
	Femenino	139 (73,5)	119(56,4%)	258 (64,5)
	Masculino	50 (26,5)	92 (43,6%)	142 (35,5)
Escolaridad				
	0 a 4	34(18)	56 (26,5)	90 (22,5)
	5 a 8	86 (45,5)	93 (44,1)	179 (44,8)
	9 a 11	40 (21,2)	33(15,6)	73(18,2)
	> 11	29(15,3)	29(13,7)	58 (14,5)
Localización del cáncer*				
	Mama	69 (36,5)	31 (14,7)	100 (25)
	Cérvix	55 (29,1)	45 (21,3)	100 (25)
	Gastrointestinal	30(15,9)	70 (33,2)	100 (25)
	Próstata	35(18,5)	65 (30,8)	100 (25)
Estadio clínico*				
	1	17 (9,5)	7 (3,6)	24 (6,5)
	2	33(18,4)	13 (6,7)	46 (12,4)
	3	53 (29,6)	49 (25,4)	102 (27,4)
	4	76 (42,5)	124 (64,2)	200 (53,8)
Quimioterapia				
	Sí	148 (78,3)	172 (81,5)	320 (80)
	No	41 (21,7)	39(18,5)	80 (20)
Radioterapia				
	Sí	106 (56,1)	114(54,0)	220 (55)
	No	83 (43,9)	97 (46,0)	180 (45)
Cirugía*				
	Sí	114(60,3)	113(53,6)	227 (56,8)
	No	75 (39,7)	98 (46,4)	173 (43,3)
Cuidados paliativos*				
	Sí	56 (29,6)	121 (57,3)	177 (44,3)
	No	133 (70,4)	90 (42.71	223 (55,8)

* Diferencias estadísticamente significativas entre los estimadores de los dos grupos

El total de pacientes de la muestra aportó 6602 meses de seguimiento; la mediana de seguimiento fue de 20 meses y los tiempos mínimos y máximos de seguimiento fueron tres días y 28 meses, respectivamente. En la cohorte se registraron 211 fallecimientos.

La mortalidad en esta cohorte tuvo una incidencia más alta en hombres, en pacientes con cáncer gastrointestinal, estadios avanzados y en tratamiento de cuidados paliativos; las menores incidencias se encontraron en pacientes con estratos socioeconómicos altos y en tratamiento quirúrgico (cuadro 2).

**Cuadro 2:** Tasas de mortalidad por categorías de las variables

Variable		Tasa	IC_95 %_
Sexo			
	Femenino	2,65	2,21 a 3,17
	Masculino	4,36	3,56 a 5,35
Estrato socioeconómico			
	1	2,65	1,99 a 3,54
	2	3,85	3,20 a 4,64
	3	3,06	2,30 a 4,05
	4 y 5	1,31	0,49 a 3,50
Años de escolaridad			
	0 a 5	3,98	3,06 a 5,17
	5 a 8	3,10	2,53 a 3,80
	9 a 11	2,65	1,88 a 3,72
	11 o más	3,06	2,13 a 4,41
Localización del cáncer			
	Mama	1,61	1,14 a 2,30
	Cérvix	2,56	1,91 a 3,43
	Gastrointestinal	5,17	4,09 a 6,53
	Próstata	4,15	3,25 a 5,29
Estadificación			
	I	1,57	0,75 a 3,29
	II	1,36	0,79 a 2,35
	III	2,85	2,15 a 3,77
	IV	4,07	3,41 a 4,86
Quimioterapia			
	No	2,82	2,063,86
	Si	3,30	2,843,83
Radioterapia			
	No	3,29	2,70 a 4,02
	Si	3,12	2,60 a 3,75
Cirugía			
	No	3,56	2,92 a 4,34
	Si	2,94	2,44 a 3,53
Cuidados paliativos			
	No	2,19	1,78 a 2,69
	Si	4,85	4,06 a 5,80

**Figura 2.**
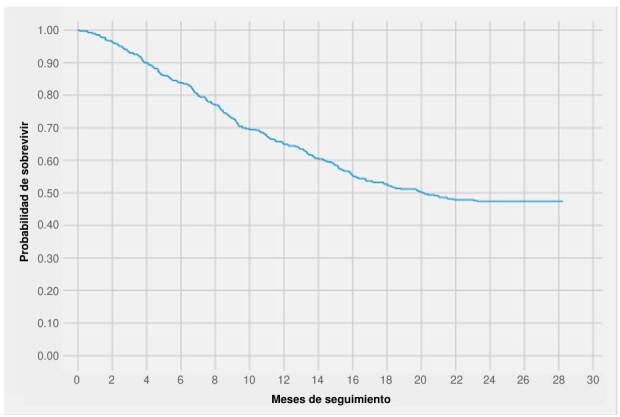
Función de supervivencia de Kaplan-Meier para la cohorte de 400 pacientes

La mediana de supervivencia en la cohorte fue de 20,2 meses; el percentil 25 de seguimiento fue de 8,5 meses y el percentil 75 no se alcanzó. La función de supervivencia de Kaplan-Meier muestra una estabilización de la probabilidad de sobrevivir luego de la mediana de supervivencia (figura 2). La función de peligro durante la cohorte muestra un incremento de la probabilidad de morir que alcanza su máximo a los 8 meses y luego disminuye progresivamente (figura 1), correspondiente a una función no monotónica creciente-decreciente.

**Figura 3:**
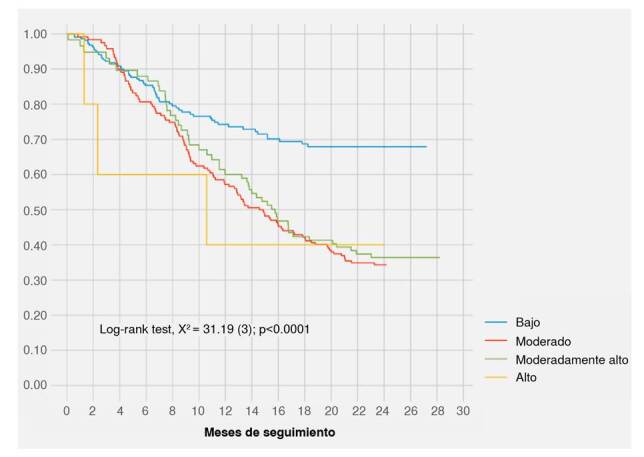
Función de supervivencia de Kaplan-Meier según niveles de soledad

La figura 3 muestra la función de supervivencia de Kaplan-Meier de acuerdo con los niveles de soledad; puede verse que la probabilidad de supervivencia se mantiene más alta en el grupo con niveles de soledad baja, en comparación con los estratos de soledad moderada, moderadamente alta y alta; esta diferencia entre las funciones de supervivencia resultó estadísticamente significativa (prueba log rank, c^2^(3) = 31,16, p < 0,0001). El grupo con niveles bajos de soledad no alcanza la mediana de supervivencia. Las tasas de mortalidad para cada uno de los niveles de soledad fueron las siguientes: bajo, 1,9 muertes por 100 pacientes-mes (IC_95%_: 1,5 a 2,5); moderado, 4,5 muertes por 100 pacientes-mes (IC_95%_: 3,7 a 5,4); moderadamente alto, 4,2 muertes por 100 pacientes-mes (IC_95%_: 3,2 a 5,5), y alto, 4,4 muertes por 100 pacientes-mes (IC_95%_: 1,4 a 13,7).

Modelo de supervivencia. Los resultados del modelo crudo utilizando la métrica de razones de peligro y tomando como valor de referencia el nivel bajo de soledad, fueron los siguientes: moderado-nivel bajo: HR = 2,33; IC_95 %_: 1,69 a 3,21; moderadamente alto-nivel bajo: HR = 2,21; IC_95%_: 1,52 a 3,23, y alto-nivel bajo: HR = 2,35; IC_95%_: 0,73 a 7,50. Los anteriores estimadores calculados con el modelo log normal arrojaron las siguientes RT: nivel moderado-nivel bajo: RT = 0,49; IC_95%_: 0,35 a 0,71; nivel moderadamente alto-nivel bajo: RT = 0,50; IC_95%_: 0,32 a 0,77; nivel alto-nivel bajo: RT =0,33; IC_95%_: 0,09 a 1,20. Lo anterior indica que, tomando como referencia el nivel bajo, los estimadores correspondientes a los niveles moderados y moderadamente altos de soledad son estadísticamente significativos.

El estimador para niveles altos tiene precisión muy deficiente, lo cual se relaciona con el tamaño de la muestra (solo cinco pacientes de la muestra presentaron niveles altos de soledad).

Los resultados del modelo multivariable se presentan en el cuadro 3. Se encuentra que, en comparación con los pacientes con niveles bajos de soledad, los pacientes con niveles moderados o moderadamente altos llegan más rápidamente a la muerte (las RT son estadísticamente significativas, habiendo ajustado por el efecto de las demás variables en el modelo).

**Cuadro 3:** Modelo de supervivencia log normal para tiempo hasta la muerte

Variables independientes		Razón de tiempo*	p**	IC_95%_
Nivel de soledad				
	Bajo	1	0,0	0,0
	Moderado	0,55	0,001	0,39 a 0,77
	Moderadamente alto	0,62	0,02	0,41 a 0,93
	Alto	1,17	0,819	0,31 a 4,42
Sexo				
	Femenino	1	0,0	0,0
	Masculino	0,85	0,549	0,50 a 1,45
	Edad	1,02	0,055	1 a 1,04
Estrato socioeconómico				
	1	1	0,0	0,0
	2	0,68	0,03	0,47 a 0,97
	3	0,90	0,66	0,57 a 1,43
	4 y 5	2,08	0,12	0,82 a 5,25
Años de escolaridad				
	0 a 5	1	0,0	0,0
	5 a 8	1,04	0,83	0,72 a 1,52
	9a 11	1,36	0,214	0,84 a 2,20
	11 o más	0,78	0,352	0,46 a 1,32
Localización del cáncer				
	Mama	1	0,0	0,0
	Cérvix	0,70	0,15	0,44 a 1,13
	Gastrointestinal	0,31	< 0,0001	0,19 a 0,51
	Próstata	0,51	0,064	0,025 a 1,04
Comorbilidad (Charlson)		0,91	0,064	0,82 a 1,01
Funcionalidad (Karnofsky)		1,03	< 0,0001	1,01 a 1,04
Estadificación del cáncer				
	I	1	0,0	0,0
	II	1,78	0,154	0,81 a 3,93
	III	1,06	0,866	0,52 a 2,17
	IV	1,09	0,826	0,51 a 2,32
Quimioterapia				
	No	1	0,0	0,0
	Sí	0,89	0,581	0,58 a 1,36
Radioterapia				
	No	1	0,0	0,0
	Sí	0,76	0,124	0,54 a 1,08
Cirugía				
	No	1	0,0	0,0
	Sí	1,59	0,005	1,15a 2,22
Cuidados paliativos				
	No	1	0,0	0,0
	Sí	0,66	0,012	0,48 a 0,91

* Valores cercanos a 0 indican un tiempo más rápido a la falla.

** La hipótesis nula que se prueba es que las razones de tiempo son iguales a 1.

Otras variables con estimadores ajustados estadísticamente significativos fueron:

- Estrato socioeconómico: la mortalidad se acelera en los pacientes con estrato socioeconómico 2, en comparación con el estrato 1.
- Localización del cáncer: en comparación con los pacientes con cáncer de mama, el desenlace de mortalidad se acelera en los pacientes con cáncer gastrointestinal.
- Funcionalidad: el incremento en el puntaje de la escala de Karnofsky retarda la presentación del desenlace de mortalidad.
- Tratamiento: el tratamiento con cirugía retrasa significativamente el desenlace de mortalidad. En aquellos pacientes en tratamiento con cuidados paliativos se acelera la mortalidad.

De acuerdo con los resultados del modelo multivariable y la magnitud de los estimadores, las variables con mayor efecto para acelerar la muerte fueron el tener cáncer gastrointestinal (RT = 0,312), seguida de niveles de soledad moderada y moderadamente alta (RTs de 0,547 y 0,616, respectivamente). Las variables con mayor efecto para retrasar la muerte fueron el tratamiento quirúrgico (RT = 1,598) y el nivel de funcionalidad (1.025).

## Discusión

Las enfermedades crónicas, como el cáncer, representan una carga económica, social y emocional significativa para la salud ^4^. Por otro lado, se ha reconocido que la soledad no deseada actúa como un factor de riesgo para la salud física y mental, teniendo implicaciones negativas en la calidad de vida y el bienestar de las personas; además, es importante destacar que la soledad no deseada no solo es una experiencia individual, sino que también tiene un componente social ^26^^,^^30^^,^^54^.

La combinación de estos dos problemas ha sido identificada como un desafío para el campo de la salud pública, lo cual justifica la necesidad de implementar políticas sociales y de salud a nivel mundial ^55^. Varias investigaciones han señalado que el experimentar sentimientos de soledad y carecer de conexiones sociales de calidad está asociado con un aumento del riesgo de mortalidad por todas las causas. Estas asociaciones se han equiparado a otros determinantes importantes de la salud que reciben una atención significativa por parte de los recursos de salud pública ^30^^,^^56^. A pesar de esto, el tema de la soledad y la falta de conexiones sociales no ha recibido la atención suficiente como determinante de la salud o indicador de riesgo comparable con otras prioridades ^56^^,^^57^.

En Colombia se han llevado a cabo varios estudios de tipo cualitativo y transversal que indican que la soledad no deseada puede tener una influencia significativa en diversos resultados en los pacientes diagnosticados con cáncer, tales como la calidad de vida, la salud mental y la adhesión al tratamiento ^58^^-^^62^. Sin embargo, hasta el momento no se había realizado un estudio longitudinal como el presente que evaluara específicamente la asociación entre la soledad y la mortalidad en pacientes colombianos diagnosticados con cáncer a lo largo de un periodo de seguimiento de dos años. Es importante destacar que en los últimos años este tema ha adquirido gran relevancia debido a los cambios sociodemográficos y su impacto en la sociedad. A continuación, se discutirán los principales hallazgos obtenidos en este estudio.

De acuerdo con los datos sociodemográficos de la muestra, se encontró una alta frecuencia de pacientes pertenecientes a estratos socioeconómicos bajos y con un bajo nivel educativo. Esto refleja las características de los pacientes que atiende el Instituto Nacional de Cancerología, pero es un factor que puede limitar la generalización de los resultados, ya que la literatura científica ha sugerido consistentemente que estas desfavorables características socioeconómicas se asocian con un mayor riesgo de mortalidad ^63^^,^^64^. Sin embargo, en el modelo multivariable no se encontró una diferencia en la aceleración de la mortalidad entre estratos socioeconómicos muy bajos, y altos o muy altos.

Las tasas más altas de mortalidad se encontraron en los grupos de cáncer gastrointestinal; estos hallazgos son consistentes con las estadísticas reportadas a nivel mundial y nacional que ubican dentro de los grupos de mortalidad más rápida a este tipo de enfermedades ^65^^,^^66^. El hecho de que este estudio se haya realizado durante la pandemia de COVID-19, posiblemente pudo haber tenido un efecto adicional sobre la mortalidad que no quedó incluido dentro de los modelos estadísticos.

Los resultados de los análisis multivariables indican que los niveles moderados y moderadamente altos de soledad se asocian con un mayor riesgo de mortalidad en los pacientes con cáncer. No se encontró esta asociación con niveles altos de soledad, pero este hallazgo se podría explicar por el bajo número de pacientes que quedó en ese estrato. Dado que éste podría ser el primer estudio en Colombia que cuantifica los niveles de soledad no deseada en los pacientes con cáncer, no tenemos antecedentes para determinar si el hallazgo de tan bajo número de pacientes con niveles altos de soledad es una característica de pacientes de este país, o es característica de los pacientes con cáncer, o puede corresponder a una debilidad del instrumento (escala de soledad de UCLA-3); aunque en una validación hecha en Colombia se encontró que el instrumento tiene adecuadas propiedades de medición ^46^, existe polémica sobre la capacidad de estos instrumentos para captar diferentes dimensiones de soledad; un estudio que revisa ocho de las escalas más frecuentemente usadas para medir soledad, incluida la escala UCLA-3, concluye que las escalas suelen tener problemas en la validez de constructo al incorporar ítems que no miden todos los componentes del constructo ^67^.

Es de anotar que la diferenciación entre estratos de soledad resulta cuestionable teniendo en cuenta las características de las funciones de supervivencia y de los estimadores de los modelos de supervivencia (los estratos correspondientes a los niveles moderado y moderadamente alto no se diferencian bien ni en los gráficos ni en los valores de los estimadores); esto supondría efectuar estudios adicionales que permitan establecer puntos de corte que muestren una mejor calidad de discriminación de las categorías. En un estudio realizado en Finlandia se reportó que, en los pacientes con cáncer, la soledad puede predecir la mortalidad por cáncer, pero que esta asociación se pierde cuando se ajusta por variables relacionadas con estilo de vida y puntajes de depresión ^35^.

En el presente estudio no efectuamos un ajuste por la presencia de depresión, aunque para algunos autores la soledad puede ser parte del mismo síndrome depresivo, caso en el cual incorporar esta variable en los modelos podría ser innecesario o incluso contraindicado. Otros estudios han propuesto que la soledad es un factor de riesgo independiente para la depresión ^68^. En los pacientes con cáncer algunos estudios establecen una relación entre depresión, estigma social, incertidumbre, apoyo familiar y calidad de vida ^69^^-^^71^; con estos planteamientos, no haber incluido variables que midan la depresión, o aspectos relacionados con ésta como los antes mencionados, puede ser una limitación del presente estudio.

Además de encontrar la asociación entre mortalidad y niveles de soledad, hubo otros hallazgos en el modelo de supervivencia: la cirugía como factor que desacelera el tiempo hasta la muerte puede relacionarse con el menor uso de esta modalidad terapéutica en los pacientes con estadios avanzados; en los pacientes con cáncer, la cirugía con intención curativa es claramente una indicación terapéutica para los pacientes con estadios no avanzados y menor riesgo de mortalidad.

El tratamiento paliativo se constituye en un factor acelerador de la muerte al no indicarse su uso en estadios tempranos ^72^.

La funcionalidad del paciente se ha descrito como factor de buen pronóstico en los pacientes con cáncer, especialmente en los de edad avanzada; además, se ha encontrado que los puntajes elevados en la escala de Karnofsky se relacionan con mejor supervivencia en pacientes sometidos a radioterapia paliativa ^73^^,^^74^.

Se encontró que el estrato socioeconómico es un factor que se relaciona con la mortalidad, ya que pertenecer a un estrato socioeconómico bajo (estrato 2) acelera el tiempo a la muerte si se compara con uno más bajo (estrato 1); este hallazgo no concuerda con lo reportado en la literatura que sostiene que mayor nivel de pobreza supone mayor riesgo de mortalidad ^75^; sin embargo, la diferencia entre estos dos estratos no es muy acentuada y, probablemente, el riesgo se deba a algún factor relacionado con barreras que pueden ser de naturaleza geográfica, económica o psicológica, lo que limita las oportunidades de detección temprana y atención adecuada, por lo que el estrato socioeconómico termina jugando un papel importante en la relación entre la soledad, el cáncer y la mortalidad.

A menudo, existe una interrelación entre la falta de recursos económicos y el sentimiento de soledad y el aislamiento social, lo que amplifica los efectos negativos en la salud de los pacientes. Además, los pacientes de estratos socioeconómicos más bajos pueden enfrentar barreras adicionales en el acceso a los servicios de salud, como la falta de transporte, la escasez de centros médicos cercanos o la imposibilidad de cubrir los costos del tratamiento; de todos modos, es importante tener en cuenta que la categorización en estratos socioeconómicos no es la mejor opción para aproximarse a las condiciones socioeconómicas de la persona pues se basa en características de la vivienda y no incluye otros aspectos relevantes; por lo cual, los hallazgos mencionados deben tomarse con cautela.

Los hallazgos del presente estudio sugieren que la soledad puede actuar como un factor de riesgo independiente y subyacente que contribuye al deterioro de la salud y la aceleración del proceso de enfermedad en estos pacientes.

Finalmente, es importante destacar que la soledad no solo afecta a nivel individual, sino que también puede estar relacionada con barreras en el acceso a los servicios de salud y el apoyo adecuado. La falta de compañía y apoyo emocional puede dificultar la búsqueda de atención médica o la adhesión al tratamiento, lo que puede retrasar la detección temprana y el inicio de terapias efectivas.

Como limitaciones del presente estudio destacamos las siguientes:

- El estrato correspondiente a niveles altos de soledad tuvo una muestra muy pequeña; esto pudo afectar la calidad de los estimadores y dificultar la exploración de un posible efecto de dosis.
- No se midieron otras covariables que pudieron haber afectado la relación encontrada entre soledad y mortalidad (por ejemplo, niveles de depresión, calidad de vida o niveles de dolor).
- Faltan estudios de validación adicionales que verifiquen que la escala capte todas las dimensiones del constructo de la soledad no deseada en pacientes con cáncer, por ejemplo, aspectos relacionados con convivencia y funcionalidad familiar.
- Algunos de los eventos incluidos dentro del desenlace “mortalidad por cualquier causa” pudieron haber sido causados por COVID19 y no tener relación con los niveles de soledad. Esto se hubiera podido manejar desde el análisis estadístico con modelos de riesgos competidores; sin embargo, no se pudo obtener la información de causa de muerte de los casos.

Como fortalezas del estudio se destaca que se tomó una muestra de pacientes que incluyó cuatro de las diferentes localizaciones de cáncer de mayor frecuencia en el país, que incorporó un diseño de cohorte con un seguimiento que permitió encontrar el número de desenlaces previsto para mantener la precisión de los estimadores, y que utilizó una estrategia de mediciones repetidas de la variable independiente que optimiza la calidad de las estimaciones, en comparación con una única medición de variables en línea de base.

En conclusión, los resultados sugieren que los mayores niveles de soledad pueden tener un efecto acelerador sobre la mortalidad de los pacientes con cáncer. La soledad tiene un efecto sobre la mortalidad que es independiente de las demás variables. Por consiguiente, el abordar la soledad como un determinante de salud en pacientes con cáncer desde una perspectiva de salud pública podría mejorar la calidad de vida, la respuesta al tratamiento y, en última instancia, reducir la mortalidad. Esto requiere una atención integral que incluya la detección, prevención y mitigación de la soledad, así como la eliminación de las barreras que dificultan el acceso a los servicios de salud.

Los resultados de este estudio brindarían una base científica sólida para respaldar la implementación de políticas de salud pública que aborden la asociación entre la soledad no deseada y la mortalidad en pacientes con cáncer.
